# Incorporating Patient and Provider Voices into the Veterans Pain Care Organizational Improvement Comparative Effectiveness Study: Informing Future Implementation

**DOI:** 10.1007/s11606-025-09639-8

**Published:** 2025-06-06

**Authors:** Hildi J. Hagedorn, Natalie Purcell, Beth M. DeRonne, Hope Salameh, William C. Becker, Karen H. Seal, Erin E. Krebs

**Affiliations:** 1 Center for Care Delivery & Outcomes Research, Minneapolis VA Health Care System, Minneapolis, MN USA; 2 Department of Psychiatry & Behavioral Sciences, University of Minnesota Medical School, Minneapolis, MN USA; 3 Center for Data to Discovery & Delivery Innovation, San Francisco VA Health Care System, San Francisco, CA USA; 4 Department of Social & Behavioral Sciences, University of California, San Francisco, San Francisco, CA USA; 5 Pain Research, Informatics, Multi-morbidities and Education Center of Innovation, VA Connecticut Healthcare System, West Haven, CT USA; 6 Section of General Internal Medicine, Yale School of Medicine, New Haven, CT USA; 7 Department of Medicine and Department of Psychiatry & Behavioral Sciences, University of California, San Francisco, San Francisco, CA USA; 8 Division of General Internal Medicine, University of Minnesota Medical School, Minneapolis, MN USA

**Keywords:** pain management interventions, implementation, qualitative research

## Abstract

**Background:**

Clinicians and healthcare systems have little evidence available to guide effective strategies to manage pain while reducing opioid use. The Veterans Pain Care Organizational Improvement Comparative Effectiveness (VOICE) trial tested two strategies to manage pain and reduce opioid use in primary care settings: interdisciplinary pain team (IPT) and pharmacist collaborative management (PCM).

**Objectives:**

This qualitative process evaluation was conducted parallel to the effectiveness trial to inform future implementation efforts.

**Design:**

Ethnographic observations and semi-structured interviews.

**Participants:**

Study staff (*n*=19), facility clinicians (*n*=37), facility clinical champions (*n*=4), and patients (*n*=32) from 10 Veterans Health Administration (VHA) facilities.

**Approach:**

Guided by the Practical Implementation Sustainability Model (PRISM), we used rapid analysis procedures to identify and categorize themes relevant to implementation. Key themes were identified for the PRISM constructs of implementation and sustainability infrastructure, organizational characteristics, organizational perspectives of the interventions, patient perspectives of the interventions, patient characteristics, and the external environment. To facilitate the development of recommendations for successful and sustainable implementation, identified themes were also mapped to the Reach, Effectiveness, Adoption, Implementation, and Maintenance (RE-AIM) outcomes, which are part of the PRISM framework.

**Key Results:**

Successful adoption required leadership support and scanning the environment for existing similar programs and interested, knowledgeable clinical champions. Implementation was supported by training in core features of the interventions, which included meaningful patient involvement in decision-making, responsiveness of the clinical team, and the longevity and intensity of the interventions. Maintenance was supported through sustained leadership support for dedicated clinical team positions and standardized roles and procedures.

**Conclusion:**

This process evaluation identified strategies to support the successful implementation and sustainment of both interventions. Implementation considerations are particularly important as sites determine which intervention(s) to adopt, given that the VOICE trial found the interventions to be similarly effective at improving pain and reducing opioid dosage.

**Supplementary Information:**

The online version contains supplementary material available at 10.1007/s11606-025-09639-8.

Chronic pain has been frequently treated with long-term opioid therapy (LTOT).^1^ Unfortunately, LTOT is associated with unrelieved pain, poor quality of life, and serious adverse events^2–10^. Guidelines recommend providing multi-modal pain care and, when opioid risks outweigh benefits, collaborating with patients on individualized opioid dose reduction.^11,12^ However, there is little evidence available to guide clinicians or healthcare systems on effective strategies to manage pain while reducing opioid use.^13–15^

The Veterans Pain Care Organizational Improvement Comparative Effectiveness (VOICE) study was a pragmatic randomized trial conducted at 10 geographically diverse Veterans Health Administration (VHA) facilities that compared two strategies for delivering patient-centered care to manage pain and reduce opioid use in the primary care setting: integrated pain team (IPT) and pharmacist collaborative management (PCM).^16^ Both interventions provided individualized pain care and opioid tapering support over 12 months. IPT required the formation of a multi-disciplinary team that included, at a minimum, a medical clinician, a mental health clinician, and a rehabilitation therapist or clinical pharmacist. The protocol required that the medical provider and mental health clinician complete the initial IPT visit together or, minimally, on the same day. The defining features of IPT included interdisciplinary team care planning, prioritization of multi-model nondrug pain management approaches, and behavioral activation visits with mental health clinicians based on motivational interviewing strategies. The defining feature of PCM was a clinical pharmacist care manager as the main point of patient contact working in collaboration with a consulting physician. The clinical pharmacist completed regular patient visits to monitor symptoms and progress towards goals, optimize medications, and facilitate referrals as indicated. Participants were not required to agree to opioid dose reduction to enroll in the study. A key feature to note is that every participating facility was required to establish an IPT and a PCM team using the staff and resources available at the facility. The primary aim of the VOICE trial was to compare the clinical effectiveness of these interventions in improving pain and reducing reliance on opioids.

A secondary pre-planned aim of the VOICE trial was to conduct a process evaluation, with the goals of identifying barriers to and facilitators of the implementation of the VOICE interventions and identifying promising strategies to enhance future implementation of effective pain care interventions. The VOICE trial found that both IPT and PCM resulted in modest improvements in pain and substantial reductions in opioid dose, with no significant differences in effectiveness between interventions.^17^ Therefore, process evaluation results take on enhanced significance for guiding clinicians and healthcare decision-makers in selecting interventions for the management of patients with chronic pain on LTOT.

## METHODS

### Overview

This implementation process evaluation used qualitative ethnographic observations and semi-structured interviews analyzed using a template-based rapid analysis technique designed to be resource-efficient and yield results comparable to traditional qualitative methods.^18–20^ Rapid qualitative analysis methods were selected given the focused research questions to be addressed and the desire to share timely feedback with operational study partners. Ethnographic observations were conducted for all study meetings relevant to the implementation, monitoring, and maintenance of the study interventions. For interviews, we sampled from populations involved in implementing, adopting, or maintaining interventions, as well as patients receiving the interventions. Data collection, starting with the all-site kickoff meeting and ending with clinician interviews, spanned 5 years from February 2017 to February 2022. The evaluation was guided by the Practical Implementation Sustainability Model (PRISM) framework.^21,22^ PRISM includes the following multilevel contextual factors relevant to program implementation throughout all stages from planning to sustainment: (1) Intervention: Organizational Perspective, (2) Intervention: Patient Perspective, (3) Recipients: Organizational Characteristics, (4) Recipients: Patient Characteristics, (5) Implementation and Sustainability Infrastructure, and (6) External Environment. PRISM also includes the RE-AIM framework implementation outcomes of Reach, Effectiveness, Adoption, Implementation, and Maintenance, which are impacted by the multilevel contextual factors.^23^ Beyond the PRISM framework, our evaluation methods allowed for novel observations and unique probes tailored to the individual participant or observed session.

The process evaluation (PE) team included an implementation scientist (HJH, PhD research psychologist), a qualitative methodologist (NP, PhD qualitative researcher), and two additional analysts (BMD, PharmD researcher; HS, BA research assistant). All PE team members had prior experience with qualitative interviewing and analysis. While the PE team members were members of the larger VOICE research team, their responsibilities on the study were specific to the PE. Due to the small number of PE team members, it was not possible to blind interviewers and analysts to study site and patient intervention condition. HJH and NP conducted observations. All members participated in conducting interviews and analysis with the exception of BMD who focused exclusively on analysis.

This study received approval from the VHA Central Institutional Review Board. All participants in ethnographic observations and interviews were either part of the study team, signed informed consent, or, in the case of some staff participants, fell under a waiver of informed consent. All interview participants understood the goals of the VOICE trial prior to interview and knew the interviewers as members of the VOICE PE team.

We followed the Consolidated Criteria for Reporting Qualitative Research (COREQ) in reporting study methods (see Appendix 1for COREQ checklist).^24^

### Ethnographic Observations

One PE team member (HJH or NP) attended each relevant meeting and took detailed, free-form notes—a form of “rapid ethnography”^25^—focused on content relevant to the PRISM constructs. Notes were handwritten or typed live and revisited immediately afterward to summarize content. Table 1 summarizes the types and numbers of meetings for which observations were completed. The all-site kickoff meeting, the site visits, and the Veteran Engagement Panel meetings were held in-person. All other meetings were virtual. Meetings ranged in size from large (e.g., the all-site kickoff meeting included over two dozen attendees) to small (e.g., site investigator planning meeting included 5 to 7 attendees).

**Table 1:** Participants, Timing, and Number of Observed Sessions for Ethnographic Observation Process Evaluation Components

Process evaluation component	Timing	Participants	Number
All-site kickoff meeting	Pre-implementation	PIs, coordinating center personnel, SIs, process evaluation team, EAB chair, PCORI representatives	1
Site visits	Pre-implementation	*For each site*: PI, coordinating center personnel, SI and coordinator, study clinicians	10
Site investigator planning calls	Pre- to early post-implementation	*For each call (1–3 sites each)*: PI(s), coordinating center representative(s), SIs	48
IPT and PCM case review meetings	Post-implementation*	*At each site and for each intervention, separately*: study clinicians, site coordinators	61
IPT support calls	Post-implementation*	IPT clinicians; facilitated by IPT lead	4
PCM support calls	Post-implementation*	PCM clinicians; facilitated by PCM lead	5
MI support calls	Post-implementation*	IPT mental health clinicians; facilitated by MI lead	16
CLP, EAB, all-site study meetings	Pre- and post-implementation	Study partners, PIs, coordinating center representatives. *In addition, for all-site*: SIs and coordinators, co-investigators	14
VEP meetings	Pre- and post-implementation	Coordinating center personnel, patient partners	2

*CLP*, clinician leadership panel; *EAB*, executive advisory board; *IPT*, integrated pain team; *MI*, motivational interviewing; *PI*, principal investigator; *PCM*, pharmacist collaborative management; *SI*, site investigator; *VEP*, Veteran engagement panel

^*^Until the final year of the study period

### Qualitative Interviews

Purposive sampling techniques were used to select participants from each target population. For small populations (e.g., site investigators), everyone was invited and all who agreed were interviewed. For larger populations, quota-based sampling was used to ensure minimally adequate inclusion of various groups^26^. For clinician interviews, the goal was to interview one clinician from each clinical role for both interventions from every site. In addition, we selected a small sample of local site champions identified as having had a significant impact on the study implementation process. For patient interviews, a minimum number of 15 each for IPT and PCM was set and the following minimum targets were used: 20% intervention non-completers; 20% women; 20% self-reporting race other than white; 20% <50 years old and 20% receiving high-dose opioids. Potential patient interviewees were approached by phone call, while potential staff interviewees were approached by email.

Multiple sets of qualitative interviews were conducted, each with a different target population and each geared at gathering data about specific aspects of the implementation process. For each set, a unique interview guide was developed that included core questions and optional probes designed to elicit respondent feedback across PRISM domains. Interviewers practiced using the guides with each other and discussed as a team how the guides functioned after first use with a participant in order to identify any wording or prompt ordering issues. Patient interviews were conducted by telephone to the patient’s home; staff interviews were conducted by telephone, video, or in-person at the staff person’s office. Only the participant and the interviewer were present. Interviews were audio-recorded and transcribed. Patient interviews were 30–60 min in length. Staff interviews were designed to take no more than 30 min. Table 2 summarizes focus, timing, target populations, and number of interviews.

**Table 2:** Focus, Target Population, Number of Interview Participants, and Timing for Each Set of Semi-Structured Interviews

Interview focus	Timing	Target population (participant number)	Number
Pre-implementation and early post-implementation	After sites started enrollment	Site investigators Study coordinators	10 9
Post-implementation and sustainment	After sites finished enrollment	Site investigators Study clinicians (2–6 per site) Site champions	7 37 4
Patient experience	After patients completed the trial	Patient participants	32

### Qualitative Data Analysis

Data collection resulted in several separate datasets including (1) ethnographic observations, (2) patient interviews, (3) research staff interviews, and (4) clinician interviews. For each dataset, a PE team member summarized information from each ethnographic note or interview into a standardized analysis template organized by PRISM constructs and, for interviews, populated relevant quotations into the template. A second PE team member analyzed a subset of notes or interviews for each dataset. Team meetings were used to discuss discrepancies and achieve consensus. Once every note or interview from a dataset was summarized on an individual template, team members read all templates and identified themes through an iterative process combining inductive and deductive content analysis.^27^ Ongoing discussions were central to the iterative elaboration and refinement of themes. One dataset at a time, themes agreed upon by all PE team members were mapped onto a master spreadsheet matrix organized into categories derived from PRISM.^21,22^ All PE team members participated in all stages of data analysis. Saturation was defined as obtaining all of the themes that were most relevant or salient to the research questions.^28^ Progress towards this criterion was assessed through regular team discussions during the interviewing and analysis process.

## RESULTS

The process evaluation results, integrating themes derived from ethnographic observations, staff, and participant interviews, are presented by PRISM constructs. Table 3 shows demographic characteristics of patient interview participants. Table 4 summarizes the qualitative themes identified for each of the PRISM constructs, the data sources from which the themes emerged, and representative supporting quotes. For each theme, the RE-AIM outcome(s) impacted by that theme are identified in bold. Reach refers to the ability of the intervention to be accessed by all patients that may benefit, Adoption refers to factors impacting the decision-making and planning process of organizations prior to implementation, Implementation refers to the ability to implement the interventions with high fidelity to the protocol, and Maintenance refers to the ability to sustain the interventions beyond the study or long term.

**Table 3:** Characteristics of Patients Who Participated in Semi-structured Interviews

Characteristic	*n*=32
Intervention/completion status, *n* (%)	
IPT completer	13 (40.6%)
IPT non-completer	3 (9.4%)
PCM completer	12 (37.5%)
PCM non-completer	4 (12.5%)
Opioid high-dose stratum, *n* (%)	8 (25.0%)
Female sex, *n* (%)	9 (28.1%)
Race other than white, *n* (%)	12 (37.5%)
Age less than 50 years, *n* (%)	7 (21.9%)

*IPT*, integrated pain team; *PCM*, pharmacist collaborative management

**Table 4:** Key Qualitative Themes, Data Sources, and Supporting Quotes

PRISM construct		
Theme and RE-AIM outcome (noted in bold)	Data sources	Supporting quotes
Implementation and Sustainability Infrastructure		
Existing positive relationships with clinical leaders eases **adoption**	Site investigator and study coordinator interviews	“I mean part of it is I have good relationships with all of these managers, so I didn’t have a problem, having all of them be supportive of the study." (Site 8, Site Investigator) “It aligned well with the Interdisciplinary Pain Committee that was going on before VOICE started and so, some of them had relationships with each other already so there was good communication and good teamwork.” (Site 4, Site Investigator) “Get your leadership commitment up front. Don’t end the conversation until leadership has fully committed—fully committed enthusiastically. If that’s not there, it’s not gonna fly.” (Site 7, Site Investigator)
Existing similar programs eased **adoption** but sometimes created challenges with **implementation**	Site investigator and study coordinator interviews; ethnographic observations	“I think what worked well is how closely it aligned with what was already happening in both the model of care in terms of scheduling and follow up but also in its alignment with clinical skills, experience, and goals of the pharmacist who was working on our VOICE PCM team.” (Site 3, Site Investigator) “I wasn’t kind of borrowing services from an existing well-oiled machine. We were really creating from scratch. So, what ended up happening is I started to partner with several services so, I’m really kind of getting buy-in from leadership in all different corners and they don’t already talk to each other.” (Site 5, Site Investigator) “Everything being very standardized was hard for existing staff, working from a protocol rather than using their judgment. Frustrating for clinicians to meet and talk about what they were supposed to be doing according to the protocol when they had been doing it for 20 years.” (Site 1, Site Investigator)
Dedicated provider time supports **adoption and maintenance**	Clinician interviews	“(Biggest barrier?) Protected time for clinical work and meetings.” (Site 1, IPT provider) “The main things that I think complicated things throughout was my participation had to come at times that I could carve out -when I didn’t already have my other clinics.” (Site 10, IPT pharmacist) “Once this study is over, we won’t be continuing because we don’t have a clinical pharmacist that can take on that role locally.” (Site 7, PCM provider)
Adaptable protocols support **implementation**	Site investigator and study coordinator interviews	It helps that the VOICE protocol had some flexibility built in so providers could practice in a manner that was relatively consistent with their existing processes and practices, so long as it fit in the broader VOICE protocol. (Site 8, Coordinator) “Another facilitator to implementation was the pragmatic focus of the research team and their openness to hearing feedback and making changes as needed. Knowing that things were not set-in-stone could relieve stress for the clinicians.” (Site 9, Coordinator)
Coordinating center supports **implementation**	Site investigator and site coordinator interviews	“But I really commend the whole [coordinating] team with how organized and thoughtful and easy to implement it was and the communication was good.” (Site 10, Site Investigator) “Having the support of the coordinating center has been key. They are there to answers questions, provide resources, run through trainings with clinicians, etc. They have a very experienced project manager who is very helpful. When implementing something so big, it helps to have someone at the center with a ‘big-picture view’ in addition to teams of workers on the ground handling different aspects of implementation. ” (Site 9, Coordinator) “All of the study materials and processes were all ironed out and they were so well established and so good and polished.” (Site 10, Site Investigator)
Recipients: Organizational Characteristics		
Local pressure to maintain access for all patients hinders **adoption**	Site champion interviews, ethnographic observations	“I’m not sure if you’re aware [city] has some of the longest waits for mental health services in the country and are certainly going to be visited by national in the coming weeks to discuss book-ability and productivity and things like that to maximize access. And so that makes it even more challenging to protect clinically mapped providers to research that affects their productivity.” (Site 4, Champion) Providers are overwhelmed with VOICE combined with usual clinical duties, no break on number of patients providers must see for usual clinical load (Site 2, Field Note) Primary Care leadership will have difficulty allocating clinicians to participate in anything that takes away from seeing patients. Huge increase in patient load over last 5 years with no growth in number of providers. (Site 8, Field Note)
Enthusiastic clinical champions support **adoption**	Site investigator interviews, ethnographic observations	“Clinical partners were supportive of the study. They thought that it was consistent with what they wanted to be doing. They thought it was consistent with what our hospital should be doing. And so, they helped really provide space. They helped provide staffing to staff up our IPT in order to release their clinicians from providing some services that they would have otherwise done.” (Site 8, Site Investigator) Although there were enthusiastic clinical champions at all sites, this certainly wasn’t true of the clinicians filling every role. At different sites, different roles were challenging to fill and keep filled, and there could be a lack of champions in entire important clinical areas (All site call, Field Note)
Staffing shortages impact **adoption and maintenance**	Site investigator, study coordinator and site champion interviews	“I think mental health was a little bit harder for them to free up their time. But the other service lines, I think, were pretty supportive.” (Site 10, Site Investigator) “Yeah. So, we had a bit of a unique situation here in [State] because we had difficulty finding a pharmacist to staff the PCM arm.” (Site 7, Site Investigator) “We were fortunate to be able to partition off some time with one of the nurse practitioners in primary care, but she had to sign off midway through. We started with three mental health providers, all of them have since transitioned to new clinical roles, either within the VA or outside the VA. We really struggled to maintain adequate engagement and support from our rehab medicine department.” (Site 3, Site Investigator)
Intervention: Organizational Perspective		
Increased **reach** of specialty pain care options	Site investigator, clinician and champion interviews, ethnographic observations	“Our patients with these complex pain needs, need this kind of attention. I can’t imagine how primary care alone would do this.” (Site 2, Site Investigator) “A patient comes in with pain on opiates and they wanna spend their whole appointment on pain and opiates and they have five other disease states I need to address. And so, being able to say, hey, we have this service to work with you and focus on your pain, that can then take care of what could’ve been a 20-minute conversation in two minutes” (Site 10, Primary Care clinician) “The patients who had some success received the kind of specialized care that they need as opposed to essentially being kind of stuck in those very hostile kind of loops they get into with the other providers or just kind of inefficient management so that’s good for the patients, for sure.” (Site 2, Champion)
Reducing burden on PCPs may improve **reach** of pain services and **adoption** and **maintenance** of interventions	Site investigator and clinician interviews, ethnographic observations	“I would say that this is the population that PCPs typically struggle with, so I think giving PCPs an alternate—a team that helps support them and the patients too, there may be patients who may be falling through the cracks, it helped get them an option. So, I think that was really, really valuable.” (Site 8, Site Investigator) “It’s sort of similar to how pharmacists have been utilized in kind of other areas, such a diabetes, hypertension, hyperlipidemia, where they are kind of the expert in the medications, and so they are better able to just focus kind of on one part of the Veteran, and then it kind of relieves the primary care provider from that aspect of it, so I think it just allows more visits than they might get otherwise. It sort of relieves a burden on the primary care provider.” (Site 8, PCM Pharmacist) “It took the burden off that primary care program for a little bit of time.” (Site 3, IPT Provider)
Clinician preference to only work with patients who were motivated for change was at odds with intent of VOICE and could impact **reach, adoption, and implementation** of interventions	Clinician interviews	“There is sort of a specific Veteran that this works well for and there’s a lot of Veterans that this wouldn’t work well for on account of their readiness to engage with all of these things” (Site 8, IPT Mental Health Provider) “For patients in VOICE, kind of hearing that the recommendation was to taper opioids, sometimes they would just elect to not have IPT take over the prescribing or not work with IPT on their prescriptions, because they would just rather not change anything. And that kind of made it difficult to explore other options.” (Site 9, IPT Medical Provider) “So that was kind of frustrating in its own regard. 'Cause they say they wanna work on it, but then, you know, it doesn’t ever go anywhere.” (Site 10, PCM Pharmacist)
Support and learning through weekly case review and interdisciplinary team meetings may improve **maintenance**	Clinician interviews	“We met weekly to coordinate our care for the veterans and I thought it was an overall positive experience. It was helpful to touch base with the other team members on a weekly basis just to see where patients were at.” (Site 10, IPT Rehabilitation therapist) “The actual teamwork that's been really nice. To have a team to talk about these patients.” (Site 1, IPT Medical Provider) “I felt supported by my pain MD, Dr. [name]. He and I work well together, and he let me be as independent as I needed and wanted. And when I needed him, I was able to reach out and say, hey, I think this patient may have some additional needs, I need your expert opinion on this, and he was there.” (Site 6, PCM Pharmacist)
Study coordinators often filled a care coordination role that would be essential for clinical **maintenance**	Clinician interviews	“With the general mental health clinic, I say, oh, call our front desk. They call our front desk, they leave a message, somebody calls them back, eventually, but there’s not one person that I can say, this is your person who schedules you. But with the VOICE Study, I can say, you know, call [Study Coordinator], she’s your person.” (Site 8, IPT Mental Health Provider) “Because a lot of it is incremental steps that go towards a better down the line future and when you miss a step, or life happens or people get cold feet, you’re kind of six months later if you didn’t have that additional support of someone reaching out.” (Site 7, PCM Medical Provider) “(Study Coordinator) held this whole thing together.” (Site 2, IPT Medical Provider)
Intervention: Patient Perspective		
Patients cited meaningful involvement in goal-setting and decision-making as impacting **effectiveness**	Veteran interviews	“He was a pretty cool guy. He answered all my questions. He wasn’t, well, you have to do this or you have to do that. It was more of a, well, how about you try to do this…. more or less he was just up front and honest.” (Site 4, Patient 5) “And so I liked the fact that it was equal discussion as far as choice, with the expertise of a pharmacist weighing in. Does that make sense? So in other words, I didn’t feel like somebody was just saying, oh, well this is what we’re going to do.” (Site 5, Patient 6)
Patients cited intensity and longevity of interventions as impacting **effectiveness**	Veteran interviews	“We talked every month. We adjusted some meds. We activated one, the Lyrica, and gradually increased that. Yeah, she was terrific. I miss talking to her.” (Site 5, Patient 2) “It was consistent. Phone calls came whether they were warranted or not, or whether they were scheduled or not. I did like the idea of just checking up on me out of the blue, that was nice.” (Site 6, Patient 1)
Patients cited responsiveness of clinicians as impacting **effectiveness**	Veteran interviews	“I really enjoyed the lines of communication, because when I’m called, I can communicate questions.” (Site 8, Patient 3) “Sometimes you can get down or if you’re feeling bad about yourself and your condition and it’s just a reminder that there’s somebody there that’s able to help you. It’s kinda like a lifeline I guess you could call it, you know, if I had any questions that I could call if I needed to. I do have the phone number.” (Site 4, Patient 6)
Recipients: Patient Characteristics		
Patients who were interested in new options viewed interventions as more **effective** than those who felt they had “tried everything” already	Veteran interviews, ethnographic observations	“I’m able to control my pain level better, so I can get up, and I can do more things now than I did before. I’m controlling my pain.” (Site 7, Patient 1) “It’s given me ideas on how to manage my daily life and how to make it better for myself for issues that I have” (Site 4, Patient 6) “I got the impression that perhaps this study was geared more towards Veterans that were either new to pain or, you know, not necessarily familiar with all of the steps that one can do.” (Site 3, Patient 4) Team notes challenges with engaging patients who have already experienced efforts by the system to reduce their opioids. (Site 3, field note)
External Environment		
Perception that VA required opioid reduction supported **adoption** but led to some conflict regarding VOICE goal	Ethnographic observations, clinician interviews	Investigator suggests that approaching primary care providers with a new pain management program as an alternative could be “a way to take the heat off you” with a difficult patient resistant to tapering. (Site 8, site visit field note) Investigator notes that there is a VA “naughty list” with regards to opioid prescribing metrics, and “you can say this is a prestigious, national quality improvement effort that is going to help us do better, that should be well received.” (Site Investigator call, field note) “If this study is going to be replicated in any kind of way or rolled out in any kind of way, I think it would be wise to make sure that anyone that's in charge that they have a similar vision [not mandating opioid reduction].” (Site 6, PCM Pharmacist)

### Implementation and Sustainability Infrastructure

Site investigators (SIs), who were recruited for their roles based on pain management expertise, research expertise, and knowledge of local clinical care processes, led local implementation efforts. They were supported by the centralized coordinating center, which provided assistance with adoption and implementation through guidance, protocols, training, resources, and clinician support. While all sites were eventually successful in establishing functional IPT and PCM teams, several factors impacted the ease and speed with which this was accomplished. SIs had to first gain agreement from all impacted clinical services to participate in the study and adopt the VOICE interventions. Soliciting agreements for adoption was facilitated by **pre-existing positive relationships with clinical leadership**, especially in primary care and mental health. Adoption was more challenging where such pre-existing relationships were lacking. Adoption was also facilitated by **existing similar pain management programs**, which made it easier to solicit agreement to participate from clinicians and clinical service lines.

Sites without existing services similar to the VOICE interventions spent more time garnering leadership support. While adoption was supported by existing similar teams, sites that adapted existing pain teams for VOICE sometimes encountered challenges with implementation as they had trouble pivoting those teams to adhere to VOICE protocols. Local site leaders and clinicians involved in existing programs were often invested in their current practices and wary of change.

**Obtaining dedicated time in each clinician’s schedule** was essential to the adoption of VOICE interventions. IPT required dedicated time from more clinicians and more logistical coordination to ensure team availability for meetings, case conferences, and concurrent initial patient visits. Therefore, adoption was more challenging for IPT than PCM.

Once sites had leadership buy-in and had identified dedicated team members, implementation was supported through planning calls with SIs and coordinators, which often included discussion about the protocol, where it was flexible and where consistency was required. The coordinating center helped sites navigate this terrain, ensuring the comparator interventions were sufficiently different from one another and reinforcing the importance of fidelity to key intervention components. **Adaptable protocols** eased implementation for sites.

**Support from the coordinating center**—including clear, manualized roles, step-by-step protocols, and on-demand assistance—was crucial to implementation. SIs, coordinators, and clinicians relied on the central coordinating center’s training and support for clinicians. Clinicians consistently reported feeling prepared to offer the VOICE interventions. Regular intervention support calls made coordinating center staff directly accessible to all study clinicians. Early on, these calls included troubleshooting of logistics, step-by-step reviews of management strategies, and resource sharing. As time went on, calls offered opportunities for protocol clarifications and best-practice sharing.

At the study’s conclusion, most sites lacked explicit plans to ensure maintenance, which was mainly driven by the inability to sustain dedicated clinician time. Some SIs planned to initiate these discussions with local leaders after study results were published. Many SIs and clinicians expected that some practices learned in VOICE would be sustained, but not necessarily the formal interventions. Overall, sites felt the ability to sustain the interventions would depend on the continued availability of motivated, well-supported staff and leadership prioritization of complex chronic pain care.

### Recipients: Organizational Characteristics

Adoption was slowed by **local pressures to maintain timely access for all patients**, which led to leaders’ reluctance to dedicate limited staff resources to what was often initially regarded as a strictly research activity. Leadership support was facilitated by highlighting the value of VOICE in addressing high-priority clinical challenges.

Partnering with **enthusiastic clinical champions** facilitated adoption through the establishment of successful teams, but identifying such champions was not possible at all facilities. **Short staffing** in key departments (e.g., primary care, mental health) and challenges related to clinicians going on medical leave, changing roles, or not having “protected time” for VOICE affected adoption and maintenance of the interventions at some sites. The roles that were challenging to fill or maintain varied across sites.

### Intervention: Organizational Perspective

Clinicians and local champions felt the VOICE study interventions **increased the reach of a range of pain treatments**, making them more accessible and more coordinated for patients. They also felt the interventions had **benefits for primary care providers**, unburdening them from managing complex chronic pain alone and exposing them to new strategies for managing pain and opioids. Primary care enthusiasm for the interventions can provide support for adoption and implementation.

VOICE clinicians felt VOICE roles were demanding because of the complex needs of the patients they were serving. Because patients were not required to agree to an opioid taper to enroll in the study, many patients did not seem ready or willing to reconsider their reliance on opioids. Generally, **clinicians preferred to work with patients who wanted to make changes** from the outset and remained motivated to do so. Clinicians provided feedback that agreement to taper should have been a requirement for eligibility. Some clinicians would have preferred to discharge unmotivated patients before the end of the 12-month intervention period. If they had the option, many clinicians would have chosen to work only with patients who expressed, up front, a willingness to make changes to their use of medications. Providers may be reluctant to adopt the interventions if these aspects are not revised. However, these are core principles of the VOICE interventions such that revising them would impact high fidelity implementation and the ability of the interventions to reach the patients most in need.

Despite the high level of complexity and variable motivation levels of the patients, clinicians were generally satisfied with their work on VOICE and identified key features that contributed to an overall positive experience. Clinicians felt well prepared to offer the VOICE interventions. They especially valued **weekly case review meetings as an opportunity to discuss challenging patients, refine care plans, and receive mutual support**. Given the larger team involved in IPT, IPT clinicians were more likely to report mutual support and learning new skills than PCM clinicians. Clinicians noted that it is difficult to participate in team meetings to discuss patients as part of standard clinical care because facility leadership views such meetings as taking time away from direct patient care; however, clinicians in both arms viewed weekly case review meetings as critically important to maintaining the interventions.

In addition to the opportunity for regular team interaction, clinicians cited site study coordinators (typically research assistants) as necessary members of their teams. Coordinators followed up with difficult-to-reach patients, rescheduled clinical visits, and facilitated case review meetings. Many clinicians thought a **designated care coordinator** would be necessary to maintain the interventions after the VOICE study.

### Intervention: Patient Perspective

Patients who completed the interventions and felt they were effective were more likely to attribute their effectiveness to the general aspects of the intervention approach, as compared to specific components of the clinical interventions. Patients in both arms felt most satisfied when they experienced **meaningful involvement in decision-making and goal-setting**, and when clinicians provided information, presented options, and respected their choices. This approach was widely cited by patients as a unique feature of their experience. As a result, most patients felt respected by their care team.

Both the **intensity and the longevity of the interventions** were valued in comparison with usual care. Patients felt VOICE provided sustained, personalized attention that many had not experienced in the past. It also facilitated relationship building. Some desired more frequent visits and would have liked to continue VOICE care beyond the intervention period.

**Responsiveness** of the VOICE team to routine communications and access to a single point of contact were important to patients. Patients at multiple, but not all, sites felt their VOICE experience was superior to their typical pain care experience because of the ease of communication with their VOICE team. The care coordination function was often identified as one of the most valuable elements of the patients’ experience and was frequently contrasted with their usual care experience.

### Recipients: Patient Characteristics

Participants who completed the interventions and found them to be most effective tended to be those who were **unhappy with their current pain treatment** due to side effects or perceived safety risks or who were **introduced to non-pharmacological behavioral pain management options** through VOICE. Veterans who were satisfied with their current pharmacological treatment, perceived that they had already tried everything or had a long history of pain care options found less value in the VOICE interventions.

### External Environment

**Providers perceived VA as requiring opioid dose reduction for all patients**. This led to frustrating interactions between providers and patients when goals were in conflict. At some sites, the VOICE interventions were promoted as an option to refer patients perceived as highly challenging elsewhere to meet these goals, therefore supporting adoption. Although VOICE shared the facility goal of reducing risky opioid prescribing, VOICE and facility approaches to accomplishing this goal were sometimes difficult to reconcile. The VOICE focus on a patient-centered approach to opioid dose reduction (without mandating reduction) as opposed to a strict reduction requirement sometimes created conflicts amid perceived organizational pressure for swift reductions. Primary care providers were sometimes frustrated when a patient’s participation in VOICE did not result in a substantial reduction in opioid dose.

## DISCUSSION

It is well recognized that successful implementation of innovations occurs in stages as indicated by a variety of implementation process models such as the Exploration, Preparation, Implementation, and Sustainment (EPIS) process model.^29,30^ The PRISM framework’s unique combination of contextual determinants of implementation with the RE-AIM outcomes allowed us to connect key barriers and facilitators of implementation to critical stages of the implementation process. Therefore, we were able to identify key lessons learned for each stage of implementation, which can provide a useful guide to implementation for other clinics or organizations wishing to implement the VOICE interventions.

In the adoption phase, when organizations are exploring and preparing for implementation, it is key to ensure support from clinical leaders of all impacted services at the start. Key messages for garnering support for adoption of the interventions include the fact that the VOICE interventions address a high-priority clinical challenge and evidence that having dedicated teams to manage patients with chronic pain and LTOT can reduce the burden of primary care providers.^31^ It is also essential that the goal of VOICE to reduce opioid doses through patient-centered, shared decision-making processes rather than mandatory reductions is clearly communicated to leaders to prevent misunderstandings that may undermine implementation and maintenance.

Once leadership support is established, facilities need to determine which VOICE intervention(s) they will implement given that both IPT and PCM resulted in modest improvements in pain and substantial reductions in opioid dose, with no significant differences in effectiveness between interventions.^17^ This decision can be facilitated by conducting a scan of existing programs to identify potentially similar programs and interested, knowledgeable clinicians who may serve as local clinical champions. Which intervention(s) to implement will be driven by local site resources. IPT is more complex in the sense that it includes a broader group of clinicians from different disciplines and requires full team meetings, which can be difficult to coordinate and schedule. However, it also provides additional team support and professional skill-building for clinicians, beyond the duo of pharmacist and consulting physician on the PCM team. This support, along with the opportunity to learn new approaches from varied disciplines, was highly valued by IPT clinicians.

Once an intervention(s) is selected, participating clinicians will need to be trained in the core features of the intervention(s). Beyond the evidence-based pharmacological and behavioral pain management interventions employed, patients’ experience of the interventions revealed that meaningful involvement in decision-making, responsiveness of clinicians, and the longevity and intensity of the interventions were core features for successful, positive patient experiences. Because working with patients who started the interventions without high motivation to change clearly presented a challenge to the VOICE clinicians, it is recommended that identified team members also engage in training in motivational interviewing or other techniques for establishing collaborative relationships with patients.

Once implemented, maintenance of the interventions can be supported through ongoing leadership support of dedicated clinical team positions to ensure that team positions are replaced when turnover occurs. Writing out local standard operating procedures designating the roles and responsibilities of all team members will assist with maintenance when team members change.

While these findings provide practical guidance to healthcare systems regarding the implementation of the VOICE interventions, they also highlight the complexities inherent in the implementation of clinical innovations which drive the continuing gap between evidence and practice.^32^ At this point in the evolution of implementation science, there is a vast literature identifying critical steps to successful implementation and effective strategies to move through those steps.^33–37^ These findings align with the lessons from this literature. However, the integration of this knowledge into healthcare systems remains scant. While there has been increasing commentary on the challenges within the field of implementation science which may be limiting its practical impact, healthcare systems must also be willing to dedicate the time and resources to bring implementation expertise into their organizations and allow clinicians the time and resources to plan for the implementation and maintenance of evidence-based practices.^38,39^

Key strengths of the process evaluation included the systematic and intentional collection of data using multiple qualitative methods with multiple participant populations at multiple times across all participating sites. The quantity and diversity of interviews and ethnographic notes provided a comprehensive, nuanced, and contextualized portrait of intervention implementation and experiences of those affected by VOICE.

Our use of the PRISM framework to structure interviews and our rapid analysis process is both a strength and a weakness. It ensured our approach was theoretically informed and evaluated a range of relevant domains; however, insofar as the frameworks structured our evaluation, they also reduced the likelihood of unexpected insights that did not fit within the framework. We attempted to mitigate this by probing beyond interview scripts, encouraging free-form note taking, and including open categories in analytic templates. In addition, the fact that the process evaluation team members were members of the larger VOICE study team, known to some of the staff members whom they interviewed, and not blind to study site or patient intervention may have introduced response bias. The process evaluation findings may also be limited in their applicability to other healthcare organizations given that the trial only included VHA facilities.

## CONCLUSION

Given the main VOICE trial outcomes showing the effectiveness of both interventions along with the process evaluation identification of potential strategies to successfully support the interventions through the stages of implementation, future research should focus on formally examining what implementation strategies facilitate the successful implementation of these interventions in VHA and other, more varied healthcare settings.

## Supplementary Information

Below is the link to the electronic supplementary material.

Supplementary file1 (DOCX 18 KB)

Supplementary file2 (DOCX 22 KB)

Supplementary file3 (DOCX 21 KB)

Supplementary file4 (DOCX 21 KB)

Supplementary file5 (DOCX 22 KB)

## Data Availability

Not applicable.
