# Supplementary material for: Incorporating Patient and Provider Voices into the Veterans Pain Care Organizational Improvement Comparative Effectiveness Study: Informing Future Implementation
Source: J Gen Intern Med. 2025 Jun 6;40(16):3920–32. doi: 10.1007/s11606-025-09639-8 (PMC12686239; doi:10.1007/s11606-025-09639-8)
Supplement: Supplementary file 2 — Supplementary file2 (DOCX 22 KB) [file 11606_2025_9639_MOESM2_ESM.docx]

# Veteran Interview Guide, VOICE Study

# Interviewer Instructions

This is a semi-structured interview guide. The interviewer may make slight modifications to the suggested language or question wording to elicit information relevant to the interviewee’s experience, and the interviewer may ask additional questions for follow-up and clarification. Not all questions will be appropriate for all interviewees. To determine which questions are appropriate, the interviewer should use his/her discretion based on the interviewee’s role/background and responses during the interview. Some questions may elicit similar information. If an interviewee has already provided adequate information to answer a specific question, the interviewer may skip that question.

# Introduction

Greet the interviewee. Thank them for volunteering for the interview. Explain the following:

1. *Who you are and why you are contacting them.*

Sample language:

*“As you know, I am part of a study team working on the VOICE study.”*

1. *The purpose of the interview.*

Sample language:

*“The purpose of our interview today is to learn more about your experience with the VOICE study, with the goal of improving care and helping other VA facilities provide the VOICE services in a way that best serves Veterans.”*

1. *How long the interview takes and reminder that participant can end at any time.*

Sample language:

*“Our interview today will take about 30 minutes, but you can feel free to stop the interview at any time, or to skip any questions you are not comfortable answering. Your participation is completely voluntary.”*

1. *That you request permission to audio-record and will keep identities confidential in sharing results.*

Sample language:

*“I am asking you permission to record our interview today so we can carefully analyze your responses. After the study, the results that we will share include themes, ideas, recommendations, quotes, and summaries. Your name will* ***not*** *be shared and we’ll be careful not to share any information that could lead others to identify you.”*

*Do you have any questions before we begin?*

*Would you like to proceed?*

*May I begin recording now?”*

During this interview, I will be asking you about your experience with VOICE services you received at the [NAME OF LOCAL SITE] VA.

# Interview Questions

1. The VOICE study assigned Veterans to one of two treatment programs, the Integrated Pain Team and Pharmacist Pain Care. In the Integrated Pain Team, Veterans worked with a medical provider and mental health therapist to set and achieve personal pain care goals. In Pharmacist Pain Care, Veterans worked with a dedicated pharmacist care manager to find more effective medications for pain and receive extra support with medication changes. Can you tell me which VOICE service you received, Integrated Pain Team or Pharmacist Pain Care?
2. How has your life and your health changed since you began working with [the Integrated Pain Team or Pharmacist Pain Care]?
   1. Have there been any changes in how you’re feeling day to day?
   2. Have there been any changes in what you’re doing day to day?
      1. Can you walk me through an example?
      2. What role, if any, did your VOICE treatment team play in helping you make these changes?
      3. What role, if any, did medication changes play in helping you make these changes?
3. Overall, how well did [the Integrated Pain Team or Pharmacist Pain Care] meet your needs as a patient?
   1. What worked well for you? (Why?)
   2. What didn’t work well for you? (Why?)
4. How would you describe your relationship and communication with your [Integrated Pain Team or Pharmacist Pain Care] providers? [Probe to ensure Veteran touches on overall relationship *and* communication.]
   1. Did any of your regular VA providers outside of the VOICE study (for example, your primary care doctor or nurse) talk to you about your participation in the VOICE study? What did they have to say?
5. How has your VOICE study experience affected your feelings about your health and your healthcare?
   1. Has your VOICE experience affected how you feel about your ability to improve your health? Why or why not?
   2. Has working with your VOICE treatment team affected how you feel about the VA healthcare system? If so, in what ways?
6. Before you were assigned to [the Integrated Pain Team or Pharmacist Pain Care], did you have an opinion about which service you hoped to receive?
   1. [If yes]: Why were you more interested in [the Integrated Pain Team or Pharmacist Pain Care]?
7. Do you have any worries or concerns about the VOICE study ending? Please tell me about them.
   1. Is there anything that might have made your transition out of [the Integrated Pain Team or Pharmacist Pain Care] smoother?
8. Looking back on your overall experience with [the Integrated Pain Team or Pharmacist Pain Care], how could we improve to better serve Veterans like you?
9. Would you recommend [the Integrated Pain Team or Pharmacist Pain Care] to other Veterans? Why or why not?
10. Is there anything else you would like to say about your experience with the VOICE study?
