# Supplementary material for: Incorporating Patient and Provider Voices into the Veterans Pain Care Organizational Improvement Comparative Effectiveness Study: Informing Future Implementation
Source: J Gen Intern Med. 2025 Jun 6;40(16):3920–32. doi: 10.1007/s11606-025-09639-8 (PMC12686239; doi:10.1007/s11606-025-09639-8)
Supplement: Supplementary file 3 — Supplementary file3 (DOCX 21 KB) [file 11606_2025_9639_MOESM3_ESM.docx]

# VOICE Clinician Stakeholder Interview Guide

# Interviewer Instructions

This is a semi-structured interview guide. The interviewer may make slight modifications to the suggested language or question wording to elicit information relevant to the interviewee’s experience, and the interviewer may ask additional questions for follow-up and clarification. Not all questions will be appropriate for all interviewees. To determine which questions are appropriate, the interviewer should use his/her discretion based on the interviewee’s role/background and responses during the interview. Some questions may elicit similar information. If an interviewee has already provided adequate information to answer a specific question, the interviewer may skip that question.

# Introduction

Greet the interviewee. Thank them for volunteering for the interview. Explain the following:

1. *Who you are and why you are contacting them.*

Sample language:

*“As you know, I am part of an evaluation team working on the VOICE study.”*

1. *The purpose of the interview.*

Sample language:

*“The purpose of our interview today is to learn more about your experience with the VOICE study, with the goal of improving the VOICE interventions and helping other facilities learn how to implement them.”*

1. *How long the interview takes and reminder that participant can end at any time.*

Sample language:

*“Our interview today will take about 30 minutes, but you can feel free to stop the interview at any time, or to skip any questions you are not comfortable answering. Your participation is completely voluntary.”*

1. *That you request permission to audio-record and will keep identities confidential in sharing results.*

Sample language:

*“I am asking you permission to record our interview today so we can carefully analyze your responses. After the study, the results that we will share include themes, ideas, recommendations, quotes, and summaries. Your name will* ***not*** *be shared and we’ll be careful not to share any information that could lead others to identify you.”*

*“Do you have any questions before we begin?”*

*“May I begin recording now?”*

# Interview Questions

1. Please tell me about your role in the VOICE study.
   1. Were you a provider for the Integrated Pain Team (IPT) or Telecare Collaborative Management (TCM) intervention?
2. Please tell me about your overall experience with the [IPT or TCM] intervention—for example, has it been positive, negative, complicated? How so?
3. I want to hear a little bit about how the [IPT or TCM] intervention was initially set up at your facility: Were there any significant issues or barriers that you encountered? Please tell me about them. (For each significant barrier: What was done to overcome that barrier?)
4. Now that you’ve had a lot of experience treating patients in the [IPT or TCM] intervention, how do you think it’s working?
   1. What’s working well and what isn’t?
   2. How well do you think the intervention is meeting the needs of the patients it serves? (Please elaborate and explain why you answered as you did.)
   3. How well do you think the intervention is meeting the needs of other clinicians in the healthcare system, for example primary care providers who have patients participating in [IPT or TCM]? (Please elaborate and explain why you answered as you did.)
5. What unique value, if any, does the [IPT or TCM] intervention bring to your healthcare system? (Please elaborate.)
6. Are there any significant challenges or concerns that have come to light since [IPT or TCM] has been operating in your healthcare system? What are they?
7. What changes, if any, would you make to the [IPT or TCM] intervention? (These can include any changes at all, from the content of the program, to how its staffed, how it’s structured, who it serves, etc.)
8. What would it take for your facility to sustain the [IPT or TCM] intervention after the study is completed? For example-- what resources? buy-in from whom?
   1. Do you think the [IPT or TCM] intervention will continue to be offered at your facility for the foreseeable future? Why or why not?
   2. Do you think it *should* be sustained? Why or why not?
9. What advice would you have for other teams trying to set up an [IPT or TCM] team?
10. Is there anything else you would like to share about the [IPT or TCM] intervention or your own experiences with it?
