# Supplementary material for: Incorporating Patient and Provider Voices into the Veterans Pain Care Organizational Improvement Comparative Effectiveness Study: Informing Future Implementation
Source: J Gen Intern Med. 2025 Jun 6;40(16):3920–32. doi: 10.1007/s11606-025-09639-8 (PMC12686239; doi:10.1007/s11606-025-09639-8)
Supplement: Supplementary file 4 — Supplementary file4 (DOCX 21 KB) [file 11606_2025_9639_MOESM4_ESM.docx]

# VOICE Site PI Interview Guide

# Interviewer Instructions

This is a semi-structured interview guide. The interviewer may make slight modifications to the suggested language or question wording to elicit information relevant to the interviewee’s experience, and the interviewer may ask additional questions for follow-up and clarification. Not all questions will be appropriate for all interviewees. To determine which questions are appropriate, the interviewer should use his/her discretion based on the interviewee’s role/background and responses during the interview. Some questions may elicit similar information. If an interviewee has already provided adequate information to answer a specific question, the interviewer may skip that question.

# Introduction

Greet the interviewee. Thank them for volunteering for the interview. Explain the following:

1. *Who you are and why you are contacting them.*

Sample language:

*“As you know, I am part of the VOICE study implementation evaluation team.”*

1. *The purpose of the interview.*

Sample language:

*“The purpose of our interview today is to learn more about your site’s experience with the VOICE study and the VOICE interventions, with the goal of helping other facilities learn from your experience.”*

1. *How long the interview takes and reminder that participant can end at any time.*

Sample language:

*“Our interview today will take about 30 minutes, but you can feel free to stop the interview at any time, or to skip any questions you are not comfortable answering. Your participation is completely voluntary.”*

1. *That you request permission to audio-record and will keep identities confidential in sharing results.*

Sample language:

*“I am asking you permission to record our interview today so we can carefully analyze your responses. After the study, the results that we will share include themes, ideas, recommendations, quotes, and summaries. Your name will* ***not*** *be shared and we’ll be careful not to share any information that could lead others to identify you.”*

*“Do you have any questions before we begin?”*

*“May I begin recording now?”*

# Interview Questions

1. Please tell me about your role as site PI in the VOICE study.
   1. Were you also a provider for the Integrated Pain Team (IPT) or Telecare Collaborative Management (TCM) intervention?
2. Please tell me about your overall experience with the VOICE study—for example, has it been positive, negative, complicated? How so?
3. I want to hear a little bit about how the process of setting up the VOICE interventions at your facility: What were the most significant issues or barriers that you encountered? Please tell me about them. (For each significant barrier: What was done to overcome that barrier?)
4. Now that your site has a lot of experience treating patients in the IPT & TCM interventions, I am interested in learning how well they are working for your site.
   1. Let’s start with the IPT intervention:
      1. From your perspective, what’s working well in delivering the IPT intervention?
      2. From your perspective, what’s not working well in delivering the IPT intervention?
         1. Are there any significant challenges or concerns that have come to light since IPT has been operating in your healthcare system? What are they?
      3. How well do you think IPT is meeting the needs enrolled patients so far? (Please elaborate.)
      4. What unique value, if any, does IPT bring to your healthcare system? (Please elaborate.)
      5. What changes, if any, would you make to the IPT intervention? (These can include any changes at all, from the content of the program, to how its staffed, how it’s structured, who it serves, etc.)
      6. Do you think the IPT intervention will continue to be offered at your facility for the foreseeable future? Why or why not?
         1. PROBE: What would it take for your facility to sustain the intervention after the study is completed? For example-- what resources? buy-in from whom?
   2. For the TCM intervention:
      1. From your perspective, what’s working well in delivering the TCM intervention?
      2. From your perspective, what’s not working well in delivering the TCM intervention?
         1. Are there any significant challenges or concerns that have come to light since TCM has been operating in your healthcare system? What are they?
      3. How well do you think TCM is meeting the needs enrolled patients so far? (Please elaborate.)
      4. What unique value, if any, does TCM bring to your healthcare system? (Please elaborate.)
      5. What changes, if any, would you make to the TCM intervention? (These can include any changes at all, from the content of the program, to how its staffed, how it’s structured, who it serves, etc.)
      6. Do you think the TCM intervention will continue to be offered at your facility for the foreseeable future? Why or why not?
         1. PROBE: What would it take for your facility to sustain the intervention after the study is completed? For example-- what resources? buy-in from whom?
5. What advice would you have for other facilities trying to set up an IPT and TCM teams?
6. Is there anything else you would like to share about the VOICE study interventions or your own experiences with them?
