# Supplementary material for: Incorporating Patient and Provider Voices into the Veterans Pain Care Organizational Improvement Comparative Effectiveness Study: Informing Future Implementation
Source: J Gen Intern Med. 2025 Jun 6;40(16):3920–32. doi: 10.1007/s11606-025-09639-8 (PMC12686239; doi:10.1007/s11606-025-09639-8)
Supplement: Supplementary file 5 — Supplementary file5 (DOCX 22 KB) [file 11606_2025_9639_MOESM5_ESM.docx]

# VOICE Stakeholder Interview Guide

# Interviewer Instructions

This is a semi-structured interview guide. The interviewer may make slight modifications to the suggested language or question wording to elicit information relevant to the interviewee’s experience, and the interviewer may ask additional questions for follow-up and clarification. Not all questions will be appropriate for all interviewees. To determine which questions are appropriate, the interviewer should use his/her discretion based on the interviewee’s role/background and responses during the interview. Some questions may elicit similar information. If an interviewee has already provided adequate information to answer a specific question, the interviewer may skip that question.

# Introduction

Greet the interviewee. Thank them for volunteering for the interview. Explain the following:

1. *Who you are and why you are contacting them.*

Sample language:

*“As you know, I am part of an evaluation team working on the VOICE study.”*

1. *The purpose of the interview.*

Sample language:

*“The purpose of our interview today is to learn your opinions about barriers and facilitators to implementing and sustaining the VOICE study interventions at your facility, as well as potential modifications to the interventions that might make them more sustainable.*

1. *How long the interview takes and reminder that participant can end at any time.*

Sample language:

*“Our interview today will take about 30 minutes, but you can feel free to stop the interview at any time, or to skip any questions you are not comfortable answering. Your participation is completely voluntary.”*

1. *That you request permission to audio-record and will keep identities confidential in sharing results.*

Sample language:

*“I am asking you permission to record our interview today so we can carefully analyze your responses. After the study, the results that we will share include themes, ideas, recommendations, quotes, and summaries. Your name will* ***not*** *be shared and we’ll be careful not to share any information that could lead others to identify you.”*

*“Do you have any questions before we begin?”*

*“May I begin recording now?”*

# Interview Questions

1. Please tell me a little bit about your role at the VA and your familiarity with the clinical interventions set up as a part of the VOICE Study.
   1. How familiar are you with the VOICE study interventions and with the teams delivering them? This includes both the Integrated Pain Team (IPT) intervention and the Pharmacist Pain Care or TCM intervention.

[*Share basic background info if familiarity is limited. For example:*]

*The VOICE study is trialing two interventions to help veterans with chronic pain optimize pain care plans that improve pain and function, enhance safety, and decrease reliance on opioid medications*

*The Integrated Pain Team intervention provides veterans with an opportunity to develop a multimodal pain care plan working with an interdisciplinary team that includes a medical provider, a mental health therapist, and one or more other providers such as a pharmacist or physical therapist. The team collaborates with the veteran to help them set and achieve personal pain care goals that may involve multiple different approaches to managing pain. The team’s mental health provider offers brief coaching calls informed by evidence-based cognitive behavioral therapy for pain.*

*In the Pharmacist Pain Care or Telecare Collaborative Management interventions, Veterans work with a dedicated pharmacist care manager to find more effective medications for pain and receive extra support from the pharmacist with medication changes. A prescribing medical provider also works closely with the pharmacist to assist with medication changes.*

- 1. Broadly speaking, how is your own work at the VA connected to or affected by these interventions?

1. Please tell me about your overall experience with the VOICE study interventions—for example, has it been positive, negative, complicated? How so? If your experience is too limited to have an opinion on this, that’s ok. We can skip to the next question.
2. In what ways, if any, were you involved in planning for or setting up the VOICE study interventions at your facility?
   1. *If involved*: Were there any significant issues or barriers that you encountered? Please tell me about them. (For each significant barrier: What was done to work through or overcome that barrier?)
   2. *If involvement was very limited*: Are you aware of any significant barriers encountered in setting up these interventions at your site? Or similar interventions?
3. In what ways, if any, were you involved in staffing, resourcing, or overseeing the VOICE study interventions? This could include authorizing (or not) the use of staff time or resources for the interventions.
   1. *If involved*: What concerns, if any, have come to light since the interventions were implemented? (For each: How have you addressed or worked through this concern?)
   2. *If involvement was very limited*: Are you aware of any challenges encountered in attempting to staff, resource, or manage these interventions at your site? (Or similar interventions?)
4. In what ways, if any, have you or your team been affected by the VOICE study interventions at your facility?
   1. If impacted:
      1. What are some of the positive impacts, if any?
      2. What are some of the negative impacts, if any?

Have you heard of any impacts, positive or negative, on your colleagues? If so, please tell me about them.

1. What unique value, if any, do you think the Integrated Pain Team (IPT) intervention and the Pharmacist Pain Care or TCM intervention may bring to your healthcare system? (Please elaborate.)

*[Probe for any differences in perspective on IPT versus TCM.]*

- 1. How well do you think they are meeting or may meet the needs of the patients they serve? (Please elaborate and explain why you answered as you did.)
  2. How well do you think the interventions are meeting or may meet the needs of clinicians in the healthcare system, for example primary care providers who have patients participating in IPT or TCM? (Please elaborate and explain why you answered as you did.)

1. How might the IPT and/or TCM interventions be modified to improve their fit with your facility’s needs and constraints? (These can include any changes at all, from the structure of the intervention, to how its staffed, who it serves, etc.)
2. Do you think the VOICE interventions will continue to be offered at your facility for the foreseeable future? Why or why not?

*[Probe for any differences in perspective on IPT versus TCM.]*

- 1. What do you think will be your facility’s primary considerations in deciding whether to continue offering the VOICE interventions after the study is completed?
  2. Who is likely to be involved in making that decision?
  3. Do *you* think the VOICE study interventions should be sustained? Why or why not?

1. What advice would you have for other facilities that are considering implementing the Integrated Pain team or Pharmacist Pain Care interventions?
2. Is there anything else you would like to share about the VOICE study interventions, including your own opinions or experiences with them?
